# The effect of training GPs in motivational interviewing on incident cardiovascular disease and mortality in people with screen-detected diabetes. Results from the ADDITION-Denmark randomised trial

**DOI:** 10.3399/bjgpopen20X101012

**Published:** 2020-02-19

**Authors:** Morten Charles, Niels Henrik Bruun, Rebecca Simmons, Else-Marie Dalsgaard, Daniel Witte, Marit Jorgensen, Bo Christensen, Helle Terkildsen Maindal, Sune Rubak, Annelli Sandbaek, Torsten Lauritzen

**Affiliations:** 1 Senior Researcher, Department of Public Health, Research Group for General Practice, University of Aarhus, Aarhus, Denmark; 2 Senior Biostatistician, Department of Public Health, Research Group for General Practice, University of Aarhus, Aarhus, Denmark; 3 Visiting Senior Researcher, Department of Public Health, Research Group for General Practice, University of Aarhus, Aarhus, Denmark; 4 Post-doctoral Researcher, Department of Public Health, Research Group for General Practice, University of Aarhus, Aarhus, Denmark; 5 Professor, Danish Diabetes Academy, Odense, Denmark; 6 Professor, Department of Public Health, Research Group for General Practice, University of Aarhus, Aarhus, Denmark; 7 Professor, Clinical Epidemiology, Steno Diabetes Center Copenhagen, Gentofte, Denmark; 8 Professor, National Institute of Public Health, Southern Denmark University, Odense, Denmark; 9 Professor, Department of Public Health, Research Group for General Practice, University of Aarhus, Aarhus, Denmark; 10 Professor, Department of Public Health, Research Group for General Practice, University of Aarhus, Aarhus, Denmark; 11 Research Manager; Health Promotion, Steno Diabetes Center Copenhagen, Gentofte, Denmark; 12 Clinical Associate Professor, Center for Pediatric Pulmonology and Allergology, Department of Child and Youth, University Hospital of Aarhus, Aarhus, Denmark; 13 Head of Unit for Cross-sectoral Collaboration and Integrated Patient Care, Steno Diabetes Center Aarhus, Aarhus, Denmark; 14 Professor, Department of Public Health, Research Group for General Practice, University of Aarhus, Aarhus, Denmark; 15 Professor, Department of Public Health, Research Group for General Practice, University of Aarhus, Aarhus, Denmark

**Keywords:** diabetes mellitus, cardiovascular diseases, general practice, motivational interviewing, randomised control trial

## Abstract

**Background:**

There is no long-term evidence on the effectiveness of training for motivational interviewing in diabetes treatment.

**Aim:**

Within a trial of intensive treatment of people with screen-detected diabetes, which included training in motivational interviewing for GPs, the study examined the effect of the intervention on incident cardiovascular disease (CVD) and all-cause mortality.

**Design & setting:**

In the ADDITION-Denmark trial, 181 general practices were cluster randomised in a 2:1:1 ratio to: (i) to screening plus routine care of individuals with screen-detected diabetes (control group); (ii) screening plus training and support in intensive multifactorial treatment of individuals with screen-detected diabetes (intensive treatment group); or (iii) screening plus training and support in intensive multifactorial treatment and motivational interviewing for individuals with screen-detected diabetes (intensive treatment plus motivational interviewing group). The study took place from 2001–2009.

**Method:**

After around 8 years follow-up, rates of first fatal and non-fatal CVD events and all-cause mortality were compared between screen-detected individuals in the three treatment groups.

**Results:**

Compared with the routine care group, the risk of CVD was similar in the intensive treatment group (hazard ratio [HR] 1.11, 95% confidence interval [CI] = 0.82 to 1.50) and the intensive treatment plus motivational interviewing group (HR 1.26, 95% CI = 0.96 to 1.64). The incidence of death was similar in all three treatment groups.

**Conclusion:**

Training of GPs in intensive multifactorial treatment, with or without motivational interviewing, was not associated with a reduction in mortality or CVD among those with screen-detected diabetes.

## How this fits in

Previous trials among individuals with type 2 diabetes have shown that motivational interviewing is associated with small, short-term improvements in some behaviours and outcomes; there is no long-term data on hard outcomes. This study shows that training of GPs in intensive multifactorial treatment, with or without motivational interviewing, was not associated with a long-term reduction in mortality or CVD among individuals with screen-detected diabetes. In a secondary analysis among those with normoglycaemia, some evidence of a spillover effect was found, leading to a reduction in incident CVD. While motivational interviewing might not be effective among individuals with diabetes, it may be a promising behaviour change technique for those at high risk of the disease.

## Introduction

Motivational interviewing is a teachable, evidence-based approach to behaviour change counselling.^1^ Drawing from several existing psychotherapy models and health behaviour change theory, motivational interviewing has been applied to the management of a wide range of behaviours and diseases, including type 2 diabetes.^1–5^ The basics of motivational interviewing can be learned and successfully applied in brief clinical interventions and the method is more effective than traditional education and advice-giving approaches in the management of chronic illness.^6^

Previous trials among individuals with type 2 diabetes have shown that motivational interviewing is associated with small, short-term improvements in some behaviours and outcomes, including dietary change, weight loss, and glycaemic control.^2,3^ However, there are documented challenges with fidelity, low-quality studies, and short-term follow-up. To the authors' knowledge, no study has evaluated the long-term effects of motivational interviewing, nor examined hard outcomes such as mortality and CVD.

The Danish arm of the ADDITION-Europe study^7^ was a trial of intensive treatment of people with screen-detected diabetes, which included training in motivational interviewing for GPs. The study aimed to assess the long-term effect of the trial intervention on incident CVD and all-cause mortality among: (i) those with screen-detected diabetes (main trial population); (ii) those found to have non-diabetic hyperglycaemia; and (iii) those who screened positive on a diabetes risk questionnaire but who were normoglycaemic on biochemical testing.

## Method

ADDITION-Denmark consisted of two phases: (i) a stepwise screening programme; and (ii) a cluster-randomised, parallel-group trial comparing the effects of intensive multifactorial treatment with routine care among individuals with screen-detected type 2 diabetes.^7,8^

### Stepwise screening programme

In brief, between 2001 and 2006, a population-based stepwise screening programme was performed among people aged 40–69 years without known diabetes in 181 general practices in Denmark. Eligible individuals were sent a diabetes risk score questionnaire^9^ with an invitation to visit their family doctor for a diabetes test (maximum 15 points) and a cardiovascular risk assessment (Heart Score^10^) if they scored ≥5 points on the risk questionnaire. Participants who attended a screening appointment underwent measurement of height, weight, blood pressure, random blood glucose (RBG), total cholesterol, and HbA1c. Individuals with an RBG ≥5.5 mmol/l or HbA1c ≥ 5.8% (40 mmol/mol) were invited to return to the practice for further testing. The World Health Organization 1999 criteria, based on a standard oral glucose tolerance test (OGTT), were used to diagnose diabetes.^11^ Participants diagnosed with type 2 diabetes were subsequently included in the treatment phase of the study.

### Randomisation

**Figure 1.**
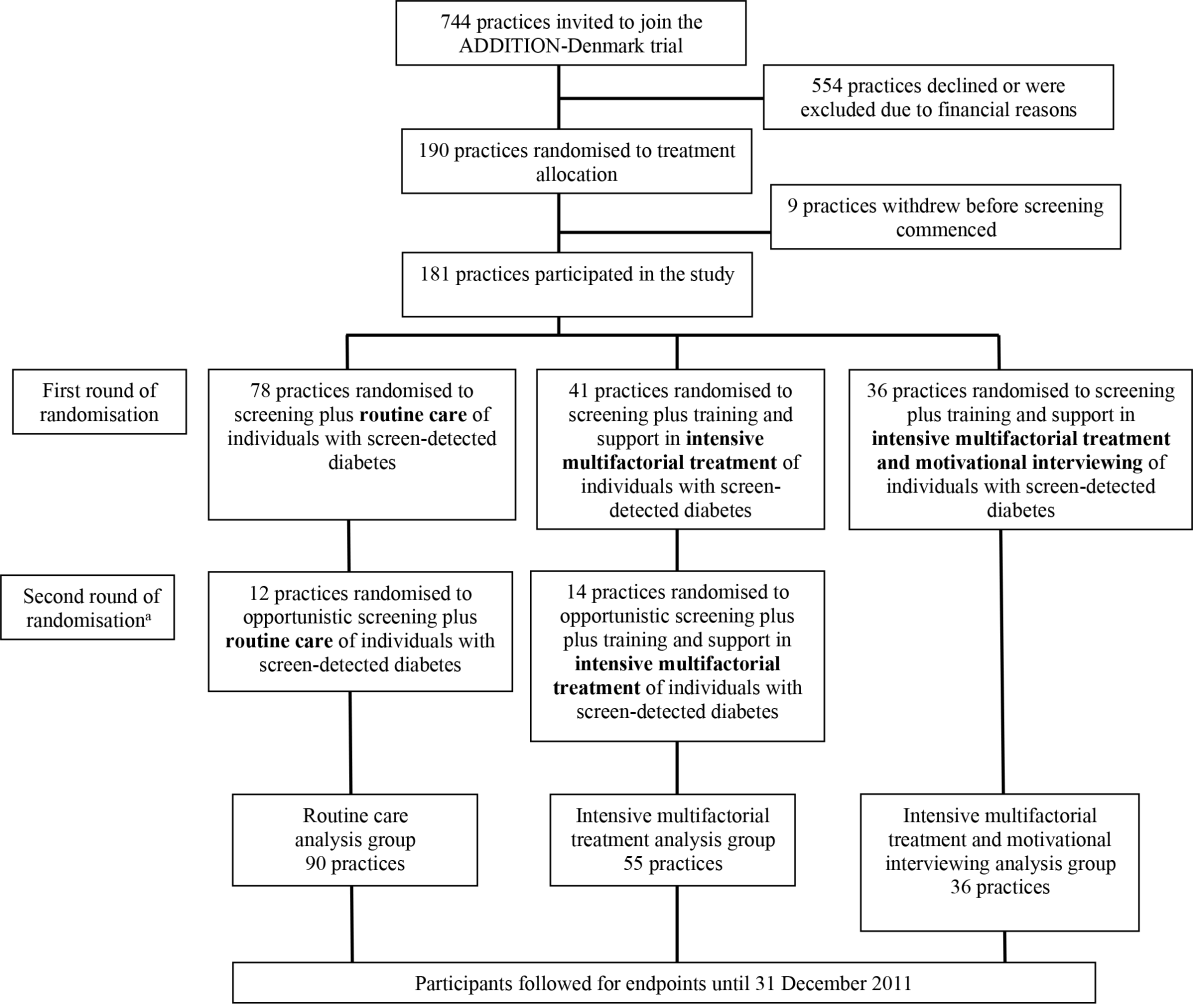
Practice and participant flow in the ADDITION-Denmark trial. ^a^Owing to restricted resources, a second round of 26 practices were randomised to opportunistic screening plus routine care or opportunistic screening plus intensive multifactorial.

The first 155 practices were randomly assigned in a 2:1:1 ratio to: (i) screening plus routine care of individuals with screen-detected diabetes (control group); (ii) screening plus training and support in intensive multifactorial treatment of individuals with screen-detected diabetes (intensive treatment group); or (iii) screening plus training and support in intensive multifactorial treatment and motivational interviewing for individuals with screen-detected diabetes (intensive treatment plus motivational interviewing group). Screening took place between 2001–2006. A second round of 26 practices conducting opportunistic screening were randomised to (i) or (ii), owing to restricted resources (Figure 1). The practices were randomly assigned by a statistician independent of the measurement team. Participants were unaware of study group allocation.

### Intervention

The characteristics of the interventions have been described previously.^8^ The study aimed to educate and support family physicians, practice nurses, and participants in target-driven management (using medication and promotion of healthy lifestyles) of hyperglycemia, blood pressure, and cholesterol, based on the stepwise regimen used in the Steno-2 study.^12^

Those practices further randomised to receive training in motivational interviewing^13^ received a 1.5 day residential course and two half-day follow-ups in the first year of the study from a single trained teacher. Miller and Rollnick’s seminal book on motivational interviewing constituted the theoretical basis of the course curriculum.^4^ Each course included 6–12 GPs and began with a short introduction to the methods of motivational interviewing. This was followed by group discussions and training, with a high level of participation in workshops and roleplays.

In the routine care (control) group, GPs were advised to follow Danish national recommendations for diabetes treatment and received no training or follow-up.

### Spillover population

As screening for type 2 diabetes identifies more people at high risk of developing diabetes than those with undiagnosed prevalent disease,^14^ the potential spillover effect of the trial intervention was assessed in a secondary analysis. Practitioners were encouraged to treat normoglycaemic individuals with a Heart Score ≥5 and those with non-diabetic hyperglycaemia, according to Danish guidelines.^15^ Individuals were identified who underwent screening as part of ADDITION-Denmark and who had non-diabetic hyperglycaemia (impaired fasting glucose [IFG] or impaired glucose tolerance [IGT]) or were normoglycaemic on biochemical testing. Normoglyacemia referred to individuals with an RBG <5.5 mmol/l and HbA1c 6% (42 mmol/mol) at the first visit or blood test, and people with fasting blood glucose <5.6 mmol/l and 2-hour blood glucose following an OGTT of <7.8 mmol/l. Information was linked about these individuals to other Danish registers using unique civil registration numbers.

### Outcomes

Participants were followed to 31 December 2011, when national registers were searched for information on vital status and incident CVD events. For death, the outcome was all-cause mortality (based on underlying cause of death). A composite of first event of cardiovascular death was used (International Classification of Diseases [ICD]-10 codes F01* and I*), non-fatal ischaemic heart disease (ICD-10 codes I20 to I25, and I46) or non-fatal stroke (ICD-10 code I60 to I69) to delineate an incident CVD event. Data on incident CVD events was gathered from the National Patient Registry, which records all inpatient and outpatient hospitalisations in Denmark. Prescription data were obtained from the Danish National Prescription Registry.

### Statistical analysis

The sample size for the trial was estimated based on the composite cardiovascular event end point (the primary end point for the trial), as previously described.^8^ The analysis was by intention to treat. Characteristics of individuals were summarised separately by trial group. Date of entry to the study was the date of invitation to screening. Individuals were censored on the date of first event following invitation for screening (for the incident CVD analysis), on death, or on the 31 December 2011 (final date of follow-up), depending on which occurred earliest. HRs comparing incident CVD events and all-cause mortality between each of the intervention groups and the routine care (control) group were estimated with a Cox proportional hazards regression model and the study accounted for clustering at the GP level. The proportional hazards assumption was tested by including a variable for treatment by time interaction in the Cox regression model (*P*>0.05). A sensitivity analysis was conducted, dropping all practices (*n* = 26) that were randomised to opportunistic screening.

In a predefined secondary analysis, the same analytical approach was used to examine the effect of the trial intervention on incident CVD and all-cause mortality among those with non-diabetic hyperglycaemia (IFG or IGT) or normoglycaemia. Following an interaction analysis, a sensitivity analysis was conducted among the normoglycaemic group, examining the same associations in individuals with a Heart Score of ≥0 to <5, ≥5 to <10, and ≥10 points. All analyses were completed using Stata (version 15.1).

## Results

### Screen-detected diabetes (trial) population (primary analysis)

Between 2001 and 2006, 1615 individuals were found with screen-detected diabetes in ADDITION-Denmark practices. Groups were well matched for baseline characteristics, with similar proportions of men (56% to 58%), and those with >15 years education (19% to 20%), as shown in Table 1. The mean age in all groups was 60 years. The proportion of individuals redeeming cardio-protective treatment was similar at baseline. There were significant increases in all classes of redeemed cardio-protective medication throughout follow-up, with the highest proportions of antihypertensive and glucose-lowering medication observed in the intensive treatment plus motivational interviewing group in 2010.

**Table 1.** Characteristics of individuals with screen-detected diabetes (*n* = 1615) and non-diabetic hyperglycaemia (*n* = 2655) in the ADDITION-Denmark study, by treatment group

	**Screen-detected diabetes**	**Non-diabetic hyperglycaemia**
**RC**(***n* = 669**)	**IT**(***n* = 524**)	**IT +** **MI**(***n* = 422**)	**RC**(***n* = 1193**)	**IT**(***n* = 863**)	**IT +** **MI**(***n* = 599**)
**Male sex,** ***n*** **(%)**	372 (55.6)	302 (57.6)	244 (57.8)	581 (48.7)	438 (50.8)	309 (51.6)
**Mean age (** **SD** **)**	59.9 (6.8)	59.4 (7.1)	59.8 (7.0)	59.8 (6.9)	60.3 (6.4)	60.5 (6.6)
**Education level,** ***n*** **(%)**						
0–10 years	246 (37.5)	203 (39.6)	151 (36.4)	475 (40.7)	331 (39.2)	199 (34.1)
10–15 years	288 (43.9)	214 (41.8)	182 (43.9)	464 (39.8)	350 (41.5)	242 (41.4)
> 15 years	122 (18.6)	95 (18.6)	82 (19.8)	227 (19.5)	163 (19.3)	143 (24.5)
Previous CVD, *n* (%)	36 (5.4)	51 (9.7)	30 (7.1)	91 (7.6)	57 (6.6)	36 (6.0)
**Redeemed anti** **hypertensive medication,** ***n*** **(%)**						
Year 2000	242 (36.2)	188 (35.9)	157 (37.2)	369 (30.9)	258 (29.9)	208 (34.7)
Year 2005	436 (66.6)	351 (68.7)	325 (79.7)	613 (52.3)	404 (47.9)	309 (53.4)
Year 2010	503 (82.7)	415 (85.9)	333 (89.8)	733 (66.3)	518 (65.9)	398 (72.2)
**Redeemed glucose-lowering medication,** ***n*** **(%)**						
Year 2000	^a^	^a^	0 (0.0)	0 (0.0)	0 (0.0)	^a^
Year 2005	173 (26.4)	177 (34.6)	181 (44.4)	27 (2.3)	23 (2.7)	22 (3.8)
Year 2010	354 (58.2)	315 (65.2)	260 (70.1)	161 (14.6)	111 (14.1)	79 (14.3)
**Redeemed lipid-lowering medication,** ***n*** **(%)**						
Year 2000	45 (6.7)	37 (7.1)	21 (5.0)	58 (4.9)	45 (5.2)	32 (5.3)
Year 2005	319 (48.7)	320 (62.6)	282 (69.1)	282 (24.0)	203 (24.1)	148 (25.6)
Year 2010	454 (74.7)	406 (84.1)	309 (83.3)	495 (44.8)	360 (45.8)	273 (49.5)
**CVD events during follow-up,** ***n*** **(%)**	116 (17.3)	98 (18.7)	93 (22.0)	221 (18.5)	143 (16.6)	120 (20.0)
**Deaths during follow-up,** ***n*** **(%)**	93 (13.9)	62 (11.8)	64 (15.2)	126 (10.6)	107 (12.4)	65 (10.9)
**HR for CVD (** **95%** **CI** **)**	–	1.11 (0.82 to 1.50)	1.26 (0.96 to 1.64)	–	0.92 (0.74 to 1.14)	1.00 (0.80 to 1.26)
**HR for all-cause mortality (** **95%** **CI** **)**	–	0.88 (0.61 to 1.27)	1.02 (0.74 to 1.39)l	–	1.23 (0.95 to 1.60)	0.88 (0.65 to 1.19)

CI = confidence intervals. CVD =cardiovascular disease. HR = hazard ratio. IT = intensive treatment. MI = motivational interviewing. RC = routine care. SD = standard deviation.

^a^Numbers too small to report (Denmark Statistics regulations).

Some percentages may not add up to expected total due to reducing cohort size over time and a small amount of missing data for some variables.

Median duration of follow-up was 8.3 years (interquartile range [IQR] 5.8 to 9.1). Compared with the routine care group, the risk of CVD was similar among the intensive treatment group (HR 1.11, 95% CI = 0.82 to 1.50) and the intensive treatment plus motivational interviewing group (HR 1.26, 95% CI = 0.96 to 1.64), as shown in Table 1. The incidence of death was similar in the three treatment groups (Table 1).

After dropping all practices (*n* = 26) that were randomised to opportunistic screening, there was no difference in the overall findings.

### Non-diabetic hyperglycaemia population (secondary analysis)

Between 2001 and 2006, 2655 individuals were found with non-diabetic hyperglycaemia following screening in ADDITION-Denmark practices. Baseline characteristics were broadly similar across treatment groups, with a mean age of 60 years and the proportion of men ranging from 49% to 52% (Table 1). The intensive treatment plus motivational interviewing group had the highest proportion of individuals with >15 years education (25%), compared with around 19% in the other two groups. The proportion of individuals redeeming cardio-protective treatment was similar at baseline. There were significant increases in all classes of redeemed cardio-protective medication throughout follow-up, with the highest proportions of antihypertensive medication observed in the intensive treatment plus motivational interviewing group in 2010.

Median duration of follow-up was 8.3 years (IQR 5.9 to 9.0). Compared with the routine care group, the risk of CVD was similar among the intensive treatment group (HR 0.92, 95% CI = 0.74 to 1.14) and the intensive treatment plus motivational interviewing group (HR 1.0, 95% CI = 0.80 to 1.26) (Table 1). The incidence of death was similar in the three treatment groups (Table 1).

### Normoglycaemic population (secondary analysis)

Between 2001 and 2006, 21 451 individuals were found with normal glycaemia following screening in ADDITION-Denmark practices. Groups were well matched for baseline characteristics, with similar proportions of men (51% to 52%) and those with >15 years education (26% to 27%) (Table 2). The mean age in all groups was 59 years. The proportion of individuals redeeming cardio-protective medication among all treatment groups was similar at baseline and throughout follow-up.

**Table 2.** Characteristics of individuals with normoglycaemia following screening in the ADDITION-Denmark study (*n* = 21 451), by treatment group

	**NGT**	**NGT and Heart Score ≥0 to <** **5**	**NGT and Heart Score ≥5 to <** **10**	**NGT and Heart Score ≥** **10**
**RC**(***n* = 10 288**)	**IT**(***n* = 6616**)	**IT +** **MI**(***n* = 4607**)	**RC**(***n* = 5042**)	**IT**(***n* = 3062**)	**IT +** **MI**(***n* = 2409**)	**RC**(***n* = 2422**)	**IT**(***n* = 1447**)	**IT +** **MI**(***n* = 1163**)	**RC**(***n* = 1670**)	**IT**(***n* = 970**)	**IT +** **MI**(***n* = 823**)
**Male sex,** ***n*** **(%)**	5250 (51.0)	3349 (50.6)	2399 (52.1)	1766 (35.0)	996 (32.5)	860 (35.7)	1610 (66.5)	966 (66.8)	759 (65.3)	1373 (82.2)	818 (84.3)	675 (82.0)
**Mean age (** **SD** **)**	59.1 (6.9)	59.0 (7.0)	59.4 (6.9)	56.0 (6.4)	56.0 (6.3)	55.8 (6.5)	62.3 (5.1)	62.4 (4.9)	62.4 (4.9)	65.1 (4.1)	65.1 (4.2)	65.2 (4.2)
**Education, *n* (%)**												
0–10 years	3191 (31.5)	2135 (32.7)	1473 (32.4)	1483 (29.8)	925 (30.5)	726 (30.5)	824 (34.5)	491 (34.3)	374 (32.7)	592 (36.2)	334 (35.1)	307 (37.9)
10–15 years	4296 (42.4)	2723 (41.7)	1832 (40.3)	2159 (43.4)	1267 (41.8)	969 (40.7)	983 (41.1)	581 (40.6)	455 (39.8)	672 (41.1)	401 (42.2)	340 (42.0)
>15 years	2640 (26.1)	1671 (25.6)	1236 (27.2)	1332 (26.8)	837 (27.6)	688 (28.9)	582 (24.4)	360 (25.1)	313 (27.4)	372 (22.7)	216 (22.7)	163 (20.1)
**Previous CVD, *n* (%)**	470 (4.6)	319 (4.8)	176 (3.8)	194 (3.8)	109 (3.6)	80 (3.3)	130 (5.4)	86 (5.9)	47 (4.0)	80 (4.8)	49 (5.1)	42 (5.1)
**Redeemed anti** **hypertensive medication,** ***n*** **(%)**												
Year 2000	2429 (23.6)	1551 (23.4)	1112 (24.1)	1180 (23.4)	727 (23.7)	543 (22.5)	606 (25.0)	362 (25.0)	300 (25.8)	405 (24.3)	223 (23.0)	217 (26.4)
Year 2005	4161 (41.0)	2599 (39.7)	1827 (40.4)	1853 (37.1)	1107 (36.4)	825 (34.6)	1036 (43.5)	602 (42.1)	501 (43.9)	837 (51.8)	473 (50.3)	422 (53.6)
Year 2010	5322 (54.9)	3416 (54.7)	2348 (54.4)	2379 (48.8)	1454 (49.3)	1101 (47.5)	1316 (58.8)	791 (58.4)	634 (58.5)	1052 (71.8)	592 (69.6)	508 (71.1)
**Redeemed glucose-lowering medication,** ***n*** **(%)**												
Year 2000	^a^	0 (0.0)	0 (0.0)	0 (0.0)	0 (0.0)	0 (0.0)	^a^	0 (0.0)	0 (0.0)	0 (0.0)	0 (0.0)	0 (0.0)
Year 2005	23 (0.2)	14 (0.2)	9 (0.2)	11 (0.2)	8 (0.3)	6 (0.3)	^a^	^a^	^a^	7 (0.4)	^a^	^a^
Year 2010	160 (1.7)	110 (1.8)	79 (1.8)	78 (1.6)	49 (1.7)	41 (1.8)	33 (1.5)	24 (1.8)	20 (1.8)	40 (2.7)	19 (2.2)	13 (1.8)
**Redeemed lipid-lowering medication,** ***n*** **(%)**												
Year 2000	383 (3.7)	232 (3.5)	142 (3.1)	162 (3.2)	98 (3.2)	60 (2.5)	111 (4.6)	70 (4.8)	40 (3.4)	76 (4.6)	44 (4.5)	35 (4.3)
Year 2005	1582 (15.6)	987 (15.1)	714 (15.8)	620 (12.4)	376 (12.4)	268 (11.3)	435 (18.3)	275 (19.2)	200 (17.5)	376 (23.3)	206 (21.9)	210 (26.7)
Year 2010	3057 (31.5)	1913 (30.6)	1354 (31.4)	1250 (25.6)	747 (25.3)	579 (25.0)	786 (35.1)	481 (35.5)	370 (34.2)	664 (45.3)	386 (45.4)	334 (46.8)
**CVD events during follow-up,** ***n*** **(%)**	1665 (16.22)	983 (14.9)	690 (15.0)	617 (12.2)	342 (11.2)	248 (10.3)	430 (17.8)	277 (19.1)	206 (17.7)	460 (27.5)	225 (23.2)	202 (24.5)
**Deaths during follow-up,** ***n*** **(%)**	825 (8.0)	527 (8.0)	396 (8.6)	235 (4.7)	155 (5.1)	118 (4.9)	244 (10.1)	134 (9.3)	114 (9.8)	281 (16.8)	165 (17.0)	144 (17.5)
**HR for CVD (** **95%** **CI** **)**	–	0.94(0.86 to 1.03)	0.87(0.79 to 0.96)	–	0.92 (0.80 to 1.06)	0.81 (0.69 to 0.94)	–	1.10 (0.93 to 1.30)	0.95 (0.78 to 1.17)	–	0.84 (0.72 to 0.97)	0.85 (0.74 to 0.99)
**HR for all-cause mortality (** **95%** **CI** **)**	–	1.05(0.92 to 1.19)	0.98(0.86 to 1.12)	–	1.12 (0.89 to 1.40)	1.00 (0.81 to 1.23)	–	0.94 (0.78 to 1.14)	0.90 (0.76 to 1.07)	–	1.05 (0.86 to 1.27)	1.01 (0.79 to 1.28)

CVD = cardiovascular disease. HR = hazard ratio. IT = intensive treatment. MI = motivational interviewing. NGT = normal glucose tolerance. SD = standard deviation.

^a^Numbers too small to report (Denmark Statistics regulations).

Some percentages may not add up to expected total due to reducing cohort size over time and a small amount of missing data for some variables

**Figure 2.**
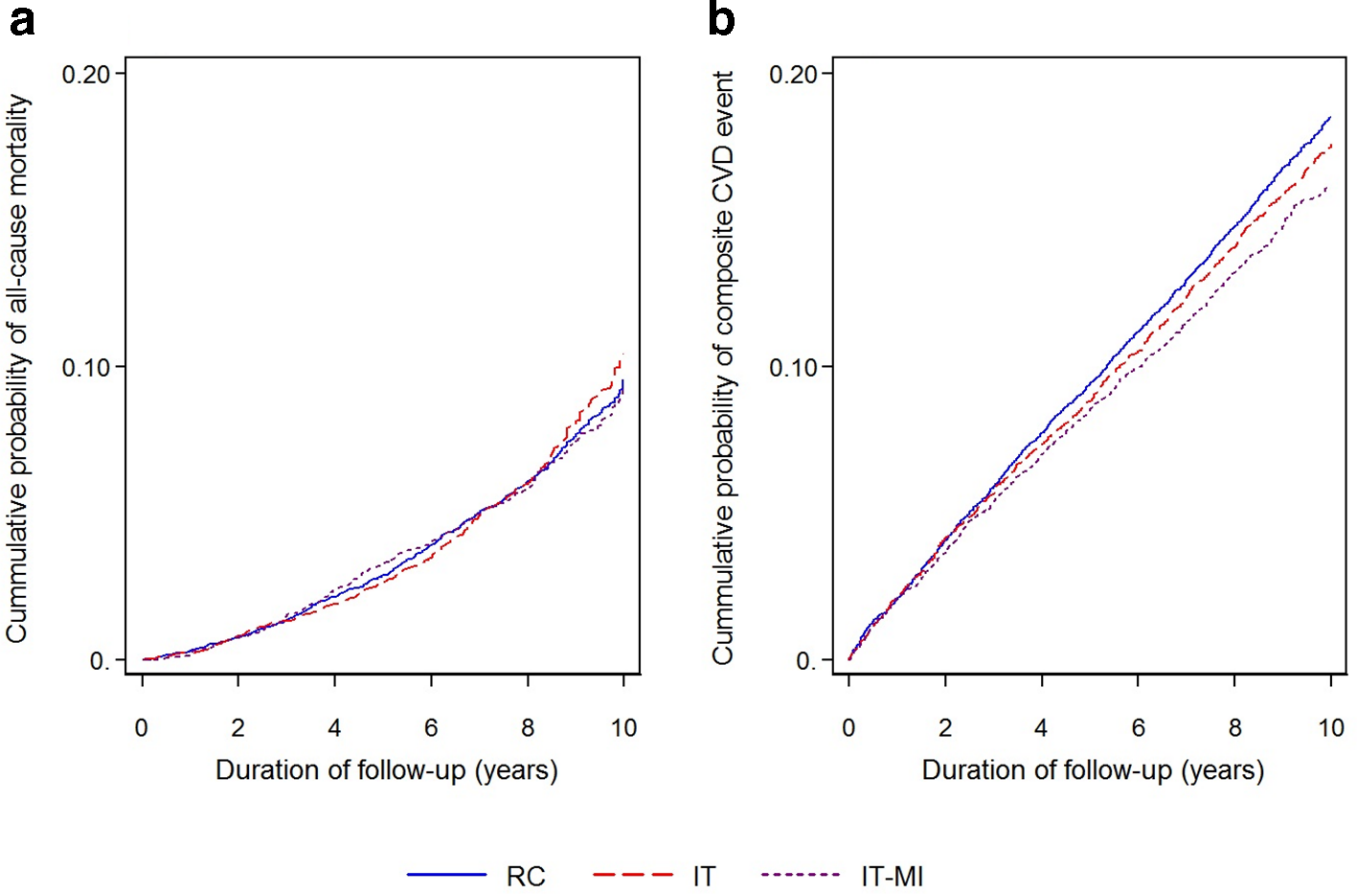
Cumulative incidence of (A) all-cause mortality and (B) incident cardiovascular disease (CVD) among individuals with normal glucose tolerance in the ADDITION-Denmark routine care (RC), intensive treatment (IT) and intensive treatment plus motivational interviewing groups (IT-MI) (2001–2011). This figure is unadjusted.

Median duration of follow-up was 8.7 years (IQR 6.2 to 9.8). Compared with the routine care group, the risk of CVD was lower among the intensive treatment group (HR 0.94, 95% CI = 0.86 to 1.03) and lower still among the intensive treatment plus motivational interviewing group (HR 0.87, 95% CI = 0.79 to 0.96), as shown in Table 2 and Figure 2. In sensitivity analyses, this trend was particularly pronounced among individuals with a low Heart Score (≥0 to <5 points), where the risk of CVD was significantly lower among the intensive treatment plus motivational interviewing group (HR 0.81, 95% CI = 0.69 to 0.94), as shown in Table 2.

The incidence of death was similar in the three treatment groups (Table 2, Figure 2) and across all levels of CVD risk.

## Discussion

### Summary

Training of GPs in intensive multifactorial treatment, with or without motivational interviewing, was not associated with a reduction in mortality or CVD over 9 years of follow-up among those with screen-detected diabetes. In a secondary analysis, there was some evidence of a spillover effect among those with normoglycaemia, leading to a reduction in incident CVD.

### Strengths and limitations

A robust, randomised controlled trial was conducted of training for motivational interviewing to improve treatment among a large group of individuals with high risk of diabetes. The Danish registry system allowed the authors to examine long-term outcomes. For those with screen-detected diabetes, trial groups were well balanced for patient level characteristics at baseline. Outcome ascertainment was robust. The Danish National Death Registry estimates 100% coverage of mortality based on death certificates, while the National Patient Registry includes 99.4% of discharges from Danish hospitals. The majority of participants were white European, the main ethnic group in Denmark, which limits generalisability to other settings.

The finding of an association between motivational interviewing and the reduction in CVD events among those with normoglycaemia is based on a secondary analysis, with all attendant challenges relating to potential bias and residual confounding. The validity of the findings could have been improved by conducting a longer-term process evaluation to examine whether changes in professional behaviour were maintained.^4^ It is not possible to gauge if the absence of effect was owing to the intervention wearing off in terms of staff behaviour reverting to previous patterns, or if it was being applied but having no effect on patient behaviour, or if both behaviours changed, but it was not sufficient to change long-term outcomes. It is known that GPs often need sustained support to be able to effectively deliver motivational interviewing.^3^ The study provided limited training in motivational interviewing for GPs (2.5 days in the first year of the intervention). Consequently, there may be potential for further improvement with repeated training. It would also have been interesting to collect long-term data on patients’ understanding of treatment, their motivation to change, and lifestyle behaviours to help establish a causal mediating link between motivational interviewing and the observed reduction in CVD risk.

### Comparison with existing literature

Previous trials among individuals with type 2 diabetes have shown that motivational interviewing is associated with small, short-term improvements in some behaviours and outcomes.^2,3^ It was unclear whether such changes might translate into long-term reductions in hard outcomes. A 1-year process evaluation was conducted, which showed that GPs receiving training reported that they changed their professional behaviour and rated motivational interviewing as more effective than ‘traditional advice-giving’ for promoting lifestyle change.^13^ People with screen-detected diabetes reported improved understanding of diabetes and beliefs concerning treatment, and were more motivated to change their lifestyle behaviour.^16^ However, there was no effect on cardiovascular risk factors or medication adherence at 1 year.^17^ It is perhaps unsurprising, therefore, that no effect on hard outcomes was found after 8 years of follow-up. The majority of this group redeemed antihypertensive (86%), lipid-lowering (80%), and glucose-lowering medication (64%). Furthermore, the practices that chose to take part in the trial, including those in the routine care group, may have been more research engaged and proactive in cardiovascular risk reduction than those that did not take part. This may have further limited the potential impact of motivational interviewing on reducing CVD risk. Indeed, the ADDITION-Denmark trial took place against a background of increasing national interest in screening and early treatment for diabetes.^8^ Similar results were observed in the secondary analysis among the non-diabetic hyperglycaemia group, where no evidence was found of a spillover effect and modest but increasing levels of cardio-protective treatment over time.

Some evidence was found for a spillover effect of motivational interviewing for people with normal blood glucose levels in the secondary analysis, extending the previous results.^18^ The trend was particularly pronounced among individuals with the lowest cardiovascular risk scores. There were much lower proportions of redeemed cardio-protective medication among this group at baseline, suggesting that there may be an early window of opportunity for motivational interviewing to support sustained behaviour change among those with normoglycaemia. This is a welcome finding, as individuals who have a positive diabetes risk score are at high risk of CVD and mortality whether or not subsequent testing shows them to have diabetes.^14^ There may have been an even greater reduction in CVD risk given the suboptimal treatment in this group compared with Danish guidelines for people at risk of CVD.^15^ For example, among people with a Heart Score ≥10, only 24% of individuals were on lipid-lowering treatment in 2005 and 46% in 2010.

### Implications for practice

Screening for type 2 diabetes inevitably identifies more people at high risk of developing diabetes and at high cardiovascular risk than those with undiagnosed prevalent disease. While there is mixed evidence on the benefits of screening^19–21^ and early treatment for those found to have diabetes^8,22–24^ results from the secondary analysis suggest that motivational interviewing may be a promising behaviour change technique for those at high risk of the disease found to be normoglycaemic at screening.

In conclusion, training of GPs in intensive multifactorial treatment, with or without motivational interviewing, was not associated with a reduction in mortality or CVD among those with screen-detected diabetes.

## Trial registration

ClinicalTrials.gov identifier: NCT00237549
